# The Combination of Aromatase Inhibitors and GH Treatment for Idiopathic Short Stature in Male Adolescents

**DOI:** 10.1210/clinem/dgaf271

**Published:** 2025-05-06

**Authors:** Yiling Cui, Qiting Zhang, Ling Hou, Xiaoping Luo

**Affiliations:** Department of Pediatrics, Tongji Hospital, Tongji Medical College, Huazhong University of Science and Technology, Wuhan 430030, China; Hubei Provincial Key Laboratory of Pediatric Genetic Metabolic and Endocrine Rare Diseases, Wuhan 430030, China; Department of Pediatrics, Tongji Hospital, Tongji Medical College, Huazhong University of Science and Technology, Wuhan 430030, China; Hubei Provincial Key Laboratory of Pediatric Genetic Metabolic and Endocrine Rare Diseases, Wuhan 430030, China; Department of Pediatrics, Tongji Hospital, Tongji Medical College, Huazhong University of Science and Technology, Wuhan 430030, China; Hubei Provincial Key Laboratory of Pediatric Genetic Metabolic and Endocrine Rare Diseases, Wuhan 430030, China; Department of Pediatrics, Tongji Hospital, Tongji Medical College, Huazhong University of Science and Technology, Wuhan 430030, China; Hubei Provincial Key Laboratory of Pediatric Genetic Metabolic and Endocrine Rare Diseases, Wuhan 430030, China

**Keywords:** short stature, puberty, aromatase inhibitors, letrozole, anastrozole, advanced bone age

## Abstract

**Objective:**

To evaluate the efficacy and safety in adolescent boys with idiopathic short stature (ISS) when treated with third-generation aromatase inhibitors (AIs), the combination of letrozole or anastrozole with recombinant human GH (rhGH), and compare adult height (AHt) augmentation following the treatment with rhGH combined with AIs or GnRH analog (GnRHa) in male adolescents with ISS.

**Method:**

We collected data from adolescent boys with ISS and a bone age ≥ 13 years who received treatment at Tongji Hospital, Huazhong University of Science and Technology from May 2017 to June 2023. Patients were allocated into the combined letrozole and rhGH group, the combined anastrozole and rhGH group, and the combined GnRHa and rhGH group based on their treatment. Three groups were matched by propensity score matching. There were 32 cases in each group. Follow-up was conducted every 3 months until AHt. Adverse events were monitored throughout.

**Results:**

The SD scores of AHt adjusted for target height in the letrozole, anastrozole, and GnRHa groups were 0.60 ± 0.28, 0.81 ± 0.34, and 0.48 ± 0.17, respectively. Compared with the letrozole and GnRHa groups, the anastrazole and rhGH combination group showed the most significant increase in AHt (*P* < .01). The abnormal monitoring indicators of the AIs group gradually returned to normal following the termination of treatment.

**Conclusion:**

For adolescent males with ISS and a bone age ≥ 13 years, the combination of AI and rhGH significantly increased the AHt. Finally, our analyses showed that anastrozole exerts more significant effects with regard to augmenting AHt with fewer adverse reactions.

## Background

Idiopathic short stature (ISS) is 1 of the most common causes of adult short stature and refers to a height more than 2 SD below the mean of the normal population of the same age, sex, and race when other causes of short stature have been ruled out. Prepubertal ISS can be treated with recombinant human GH (rhGH). Due to the small residual growth potential of adolescents with ISS and advanced bone age, rhGH treatment for the augmentation of adult lifelong height is very limited (1-3). For those who have entered puberty and have not yet reached the lowest level of the same age (<2 SD), it is necessary to delay the progression of bone age to achieve an increased adult height (AHt). At present, GnRH analog (GnRHa) is often used to delay the maturation of bone age in adolescents with ISS, which can inhibit the hypothalamic-pituitary-gonadal axis to reduce blood sex hormone levels. However, the specific efficacy of GnRHa in terms of AHt augmentation for adolescent ISS boys with advanced bone age has yet to be fully elucidated (4, 5). Furthermore, GnRHa is expensive and requires injection; therefore, some families are unable to accept this form of treatment (6). Third-generation aromatase inhibitors (AIs), especially letrozole and anastrozole, have been shown to increase predicted AHt (PAH) in children with ISS. Research indicates that the long-term use of AIs, combined with rhGH, can inhibit the synthesis of estrogen, delay the progression of bone age, and promote growth effects, thus helping to augment the AHt of children (7-12). However, the use of AIs to treat short stature in pubescent boys remains off-label (13), and there is very little direct comparative information relating to the therapeutic efficacy and safety of letrozole and anastrozole for children.

In this study, we compared AHt augmentation following the treatment of male adolescents with ISS and a bone age ≥13 years with rhGH combined with AIs or GnRHa and monitored our patient cohort for adverse reactions. Our findings provide reference guidelines for the application of AIs in the clinical treatment of male adolescents with ISS and advanced bone age.

## Materials and Methods

### Subjects

Studies were approved by the Ethics Committee of Tongji Hospital, Tongji Medical College, Huazhong University of Science and Technology (TJ-IRB20240636). We obtained informed written consent from patients' parents before treatment. Our analysis included 96 adolescent males with short stature who were treated at the Department of Pediatrics Tongji Hospital, Tongji Medical College, Huazhong University of Science and Technology between May 2017 and June 2023. The inclusion criteria were as follows: male; aged ≥ 12 years with a bone age ≥ 13 years; clear sexual development (Tanner stage II and above, with a testicular volume ≥ 4 mL); height < 2 SD of the mean height of the same age or <2 SD of the median height of the parents (−2 SD). Patients were excluded for the following reasons: endocrine, genetic, metabolic, and chronic organic diseases or systemic infections, immunodeficiency, and mental illnesses; diseases of the skeletal system (such as achondroplasia, limb asymmetry, or scoliosis); and poor compliance.

### Study Design and Groupings

The primary outcome measure of the study was AHt, and secondary outcome measures were the difference of PAH for each treatment, hormone levels, and height velocity (HV). Patients were allocated into specific groups based on their treatment, and rhGH doses were the same in the 3 groups (rhGH 0.05 mg·kg^−1^·day^−1^): (1) letrozole + rhGH: letrozole 2.5 mg/day; (2) anastrozole + rhGH: anastrozole 1 mg/day; (3) GnRHa + rhGH: 3.75 mg once every 4 weeks.

### Follow-up and Analytical Indicators

Every 3 months during the treatment period, we monitored height, HV, body weight, secondary sexual characteristics, organ function, blood lipids, insulin (RRID:AB_2756878), ACTH (RRID:AB_2783635), cortisol (RRID:AB_2810257), GH (RRID:AB_2909498), IGF-1 (RRID:AB_2756880), testosterone (RRID:AB_2905661), estradiol (RRID:AB_2892997), and gonadotropins LH (RRID:AB_2756388) and FSH (RRID:AB_2750983). Every 6 months, we acquired bone age films to evaluate PAH. We performed testicular ultrasonography and monitored bone mineral density by dual x-ray absorptiometry every year. In addition, height, body weight, and secondary sex characteristics were reviewed every 6 months to 1 year after treatment.

Bone age was evaluated by the Tanner–Whitehouse-3 methods (14). PAH was calculated by the Bayley–Pinneau method (15). The SD score of height for bone age was expressed as HtSDS_BA_. Target height was defined as [father's height (cm) + mother's height (cm) + 13]/2. According to the target height correction, the SD score for a AHt was defined as (AHt – target height)/6.1.

Treatment was discontinued for the following reasons: (1) the patient met the PAH criteria, while considering target height and growth velocity (an increase of height < 1 cm over 3 months); (2) the patient reached near-AHt (defined as growth velocity < 2 cm per year); and (3) the patient experienced serious adverse events, such as liver and kidney dysfunction or fractures.

### Statistical Analysis

Statistical analyses were performed using SPSS 19.0 software. Data before and after treatment were compared using Wilcoxon signed-rank test or paired *t*-test. The comparisons of 2 or more groups were carried out using the Kruskal–Wallis H test or 1-way ANOVA. Significant differences were identified as *P* < .05.

## Results

**Figure 1.**
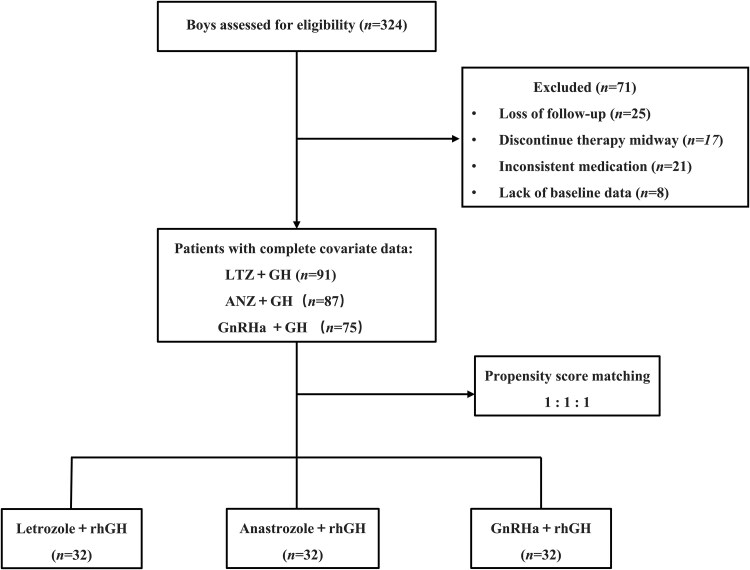
The flowchart of patients’ screening.

There were 253 patients with complete covariate data who met the inclusion and exclusion criteria of this study, including 91 cases in the letrozole combination group, 87 cases in the anastrozole combination group, and 75 cases in the GnRHa combination group. There were 32 cases in each group after propensity score matching in a 1:1:1 ratio (Fig. 1). There were no statistically significant differences in age, bone age, height-for-age SD score, bone age-for-height SD score, testicular volume, target height, and PAH when compared between the groups prior to treatment (*P* > .05) (Table 1). All patients received treatment for more than 18 months, and follow-up was conducted until AHt (Table 2).

**Table 1.** Comparison of baseline characteristics of study participants

Group	n	Age (year)	Bone age (year)	Height	ΔHtSDS_CA_	ΔHtSDS_BA_
Letrozole + rhGH	32	13.2 ± 0.89	14.0 ± 0.53	150.9 ± 2.56	−1.25 ± 0.88	−2.37 ± 0.86
Anastrozole + rhGH	32	13.0 ± 0.97	13.9 ± 0.59	151.0 ± 2.52	−1.32 ± 0.96	−2.31 ± 0.54
GnRHa + rhGH	32	12.9 ± 1.08	13.8 ± 0.67	151.2 ± 2.75	−1.28 ± 0.83	−2.29 ± 0.59
F	—	1.98	2.59	1.22	0.83	1.64
*P*	—	>0.05	>0.05	>0.05	>0.05	>0.05

“—” indicate no relevant data.

ΔHtSDSCA is the difference in SD scores for height by chronological age. ΔHtSDSBA is the difference in SD scores for height by bone age

Abbreviations: GnRHa, GnRH analog; rhGH, recombinant human GH.

**Table 2.** Comparison of bone age and AHt after treatment

Group	n	Course (year)	Δ BA/Δ CA	ΔPAH	AHtSDS_THt_	AHt (cm)
Letrozole + rhGH	32	2.1 ± 0.5	0.43 ± 0.16	9.77 ± 1.72	0.60 ± 0.28	171.8 ± 2.0
Anastrozole + rhGH	32	2.2 ± 0.3	0.59 ± 0.24	11.06 ± 2.33	0.81 ± 0.34	173.2 ± 2.3
GnRHa + rhGH	32	2.5 ± 0.3	0.62 ± 0.22	8.09 ± 1.61	0.48 ± 0.17	170.4 ± 1.3
F	—	—	13.70	38.87	19.77	28.40
*P*	—	—	<0.01	<0.01	<0.01	<0.01

“—” indicate no relevant data.

Δ BA/Δ CA is the bone age difference/chronological age difference. ΔPAH is the difference of predicted AHt after treatment. AHtSDS_THt_ is the SD score of the final AHt adjusted for genetic target height.

Abbreviations: AHt, adult height; GnRHa, GnRH analog; rhGH, recombinant human GH.

### Changes in Bone Age, PAH, and Final AHt

**Figure 2.**
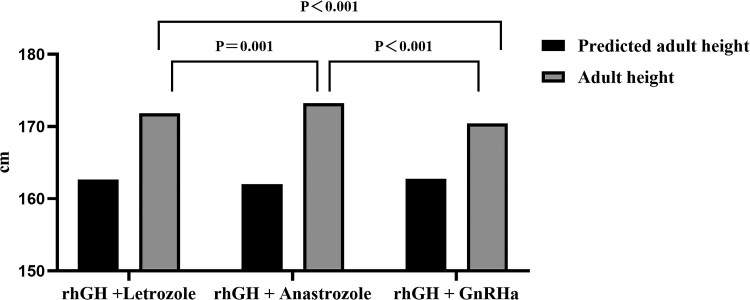
Comparison of AH in 3 groups; the improvement of AH in rhGH + AI group was better than that in rhGH + GnRHa group (170.4 ± 1.3 cm), and the difference was statistically significant (*P* < .05). The AH of the anastrozole group (173.2 ± 2.3 cm) was higher than that of the letrozole group (171.8 ± 2.0 cm). AH in all 3 groups was higher than baseline predicted AH (*P* < .001). Abbreviations: AH, adult height; GnRHa, GnRH analog; rhGH, recombinant human GH.

There were significant differences in bone age difference/chronological age difference, SD score of AHt corrected by target height, PAH difference, and AHt in the rhGH and AIs combination group (*P* < .05 or *P* < .01) compared to the rhGH and GnRHa combination group at treatment completion (Table 2). When considering the combination of rhGH and AIs at treatment completion, the bone age difference/chronological age difference in the rhGH and letrozole combination group was 0.43 ± 0.16 cm; this was significantly lower than that in the anastrozole group (0.59 ± 0.24 cm; *P* < .05), thus suggesting that letrozole may be more advantageous for controlling bone age. The PAH difference in the rhGH and anastrozole combination group (11.06 ± 2.33 cm) was significantly higher than that in the letrozole group (9.77 ± 1.72 cm; *P* < .01). Compared with letrozole and GnRHa, the anastrozole and rhGH combination group showed the most significant increase in terms of AHt (*P* < .01) (Fig. 2). When followed up to adulthood, we found that the ability of AIs to augment AHt was significantly better than that of GnRHa, and that the AHt in the anastrozole group (173.2 ± 2.3 cm) was significantly higher than that in the letrozole group (171.8 ± 2.0 cm; *P* < .05). Collectively, our results suggest that the anastrazole and rhGH combination group is more effective for the treatment of adolescents with ISS and a bone age ≥ 13 years than when combined with letrozole.

### Comparison of Growth Velocity After 18 Months of Treatment

**Figure 3.**
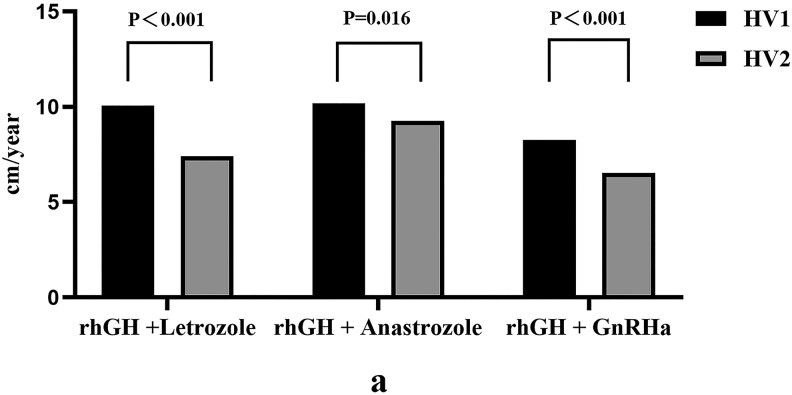
Comparison of the HV in 3 groups. The HV of the anastrozole group remained at a relatively high level during the treatment; the HV of the letrozole group decreased in the second year compared to the first year; the HV in the second year (6.76 ± 1.03 cm/year) in the GnRHa group significantly decreased compared to HV in the first year 1(8.43 ± 0.76 cm/year), showing a marked downward trend (*P* < .01). Abbreviations: HV, height velocity.

Growth velocities at 18 months were evaluated using data corresponding to the shortest treatment for the three groups. The HV in the first year for the rhGH and letrozole combination group was 9.24 ± 2.03 cm/year; this differed insignificantly from the HV in the first year in the rhGH and anastrozole combination group (10.13 ± 1.89 cm/year) (*P* > .05). The HV in the letrozole group was lower in the second year when compared to the first year but was still higher than that in the rhGH and GnRHa combination group (*P* < .01). The HV in the rhGH and anastrozole combination group remained at a relatively high level during treatment. (Fig. 3)

### Incidence of Adverse Events During Treatment

Several adverse reactions were recorded during treatment, including hyperandrogenemia, neuropsychiatric symptoms (drowsiness or memory decline), low high-density lipoproteinemia, joint pain, hyperinsulinemia, and hyperuricemia. With the exception of joint pain and hyperinsulinemia, the incidence of other adverse events in the rhGH and AIs combination group was significantly different from that of the GnRHa combination group (*P* < .05). The combination GnRHa and rhGH group exhibited a better overall safety profile than the AIs group.

**Figure 4.**
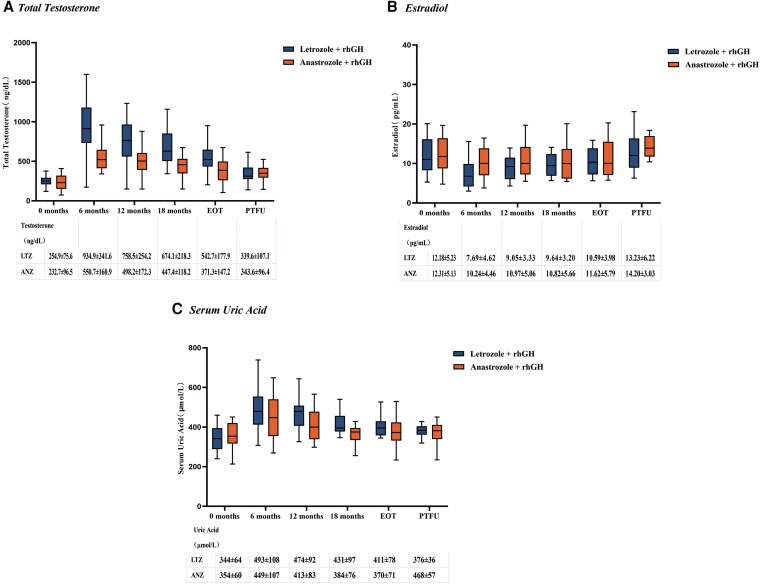
Serum testosterone, estradiol, and uric acid during treatment and follow-up in adolescent boys. The mean levels of serum testosterone (A), estradiol (B), and uric acid (C) at baseline; 6, 12, and 18 months of treatment; end of treatment; and posttreatment follow-up to 6 months.

The incidence of neuropsychiatric symptoms (drowsiness or difficulty falling asleep, memory decline), low high-density lipoproteinemia, and hyperuricemia in the rhGH and letrozole combination treatment showed significant differences when compared to the anastrozole combination group (*P* < .05) (Table 3). During treatment, the mean testosterone concentration in the rhGH and letrozole combination group was 791.39 ng/dL, which was significantly higher than the anastrozole combination group (502.15 ng/dL). Following the termination of treatment for 6 months, the abnormal monitoring indicators gradually returned to normal (Fig. 4A). In contrast, serum estradiol concentrations remained stable throughout the treatment regimen (Fig. 4B). The mean serum uric acid concentration in the rhGH and letrozole combination group was 454.36 μmol/L (Fig. 4C), which was significantly higher than the anastrozole combination group (*P* < .05).

**Table 3.** Comparison of adverse events of study participants

Group	n	Hyperandrogenism	Memory impairment	Low HDL cholesterol	Arthralgia	Hyperinsulinemia	Hyperuricemia
Letrozole + rhGH	32	13 (40.6)	7 (21.9)	10 (31.3)	5 (15.6)	2 (6.3)	9 (28.1)
Anastrozole + rhGH	32	8 (25.0)	2 (6.3)	5 (15.6)	3 (9.4)	2 (6.3)	2 (6.3)
GnRHa + rhGH	32	0 (0.0)	0 (0.0)	0(0.0)	3 (9.4)	1 (3.1)	0 (0.0)
χ^2^	—	15.73	8.73	11.85	0.83	0.61	12.58
*P*	—	<0.01	<0.01	<0.01	>0.05	>0.05	>0.01

“—” indicate no relevant data.

Abbreviations: GnRHa, GnRH analog; HDL, high-density lipoprotein; rhGH, recombinant human GH.

This study did not identify any patients with scoliosis, osteoporosis, or fractures. In the AIs combination group, baseline lumbar spine Z-scores averaged 0.53 ± 0.32 and decreased to 0.14 ± 0.31 at the end of treatment (*P* < .05); these were always within the normal range.

## Discussion

The contribution of adolescent height gain to final AHt is approximately 15% to 20% (16). After entering puberty, and with the interaction of sex hormones and GH, there is an acceleration in height growth. One of the major problems faced by adolescent children with ISS is limited growth potential. If height cannot be increased before the onset of puberty (usually within 2 SD), then most of these patients cannot catch up effectively during the limited window of growth during puberty, thus resulting in a short stature in adulthood, with AHt lagging behind by 2 SD (1).

In this study, we used a combination of rhGH and AIs (letrozole and anastrozole), rhGH, and GnRHa to treat boys with ISS and a bone age ≥ 13 years who had entered puberty. Our analyses showed that HV decreased after six months of treatment with GnRHa when combined with rhGH, and that the HV fell significantly in the second year when compared with the first year but did not decelerate excessively (an excessive decline in HV below 4 cm/year). Although HV of the letrozole and rhGH combination group also exhibited a gradual decreasing trend, the HV was always higher than that in the combined GnRHa group. The HV of the anastrozole and rhGH combination group always remained high (Fig. 3). The HV of the GnRHa group quickly declined and remained at a relatively low level, which was consistent with other studies (17). In terms of bone age control, letrozole was significantly superior to anastrozole in delaying bone age progression, especially at the very end of puberty in adolescents with ISS. Only letrozole significantly slowed down bone aging and gained more valuable growth time for children with ISS at the very end of puberty. However, previous research has shown that the action of IGF-1 will also be reduced in the growth plate as a result of the higher inhibitory potency of letrozole (18).

Although some studies have shown that AIs play a positive role in improving the height of adolescent children, it remains controversial as to whether AIs can increase the AHt of boys with ISS. Zegarra et al (19) evaluated the PAH of 79 pubertal males with short stature; after treatment with anastrozole or letrozole for 2 to 3 years, neither PAH nor height SDS increased minimally, which demonstrated that the limitation of AI as monotherapy for growth promotion in adolescent males with ISS. Hero et al (8) treated 31 boys with ISS aged 9.0 to 14.5 years with letrozole (2.5 mg/day) or placebo for 2 years in a double-blinded and placebo-controlled study. In letrozole-treated boys, PAH increased by an average of 5.9 cm, and height SD score increased by 0.7 SD relative to bone age; there was no change in boys who received the placebo. In another study, Mauras et al (9) treated adolescent boys with ISS with AIs and rhGH for 24 to 36 months; the final height gain of these boys was 22.5 (1.4) cm, which was higher than those treated with AIs or rhGH alone. Similar to the findings of most studies (7-11), the data in this study support the conclusion that the combination of AIs with rhGH can significantly augment AHt in adolescent males with ISS and advanced bone age. We found that the combination of rhGH and AIs significantly increased AHt when compared with GnRHa. In the present study, the AHt of children in the combined GnRHa and rhGH treatment group lagged behind that in the combined AIs and rhGH treatment group, and the HV decreased significantly during treatment. For ISS children with a bone age > 13 years, the effect of GnRHa as monotherapy for growth augmentation is limited and must be combined with rhGH treatment for at least 2 years to induce a significant increase in AHt (20, 21). Notably, we compared the efficacy of letrozole and anastrozole treatment during childhood. The combination of anastrozole and rhGH exhibited the most significant AHt augmentation (Fig. 2); the average AHt was 173.2 ± 2.3 cm. In a recent study, Gürkan Tarçın et al (22) evaluated retrospectively the effect of anastrozole on final height gains in 24 pubertal boys receiving rhGH. The average AHt of patients who received anastrozole combined with rhGH treatment for more than 2 years was 173.1 cm. This observation is in agreement with our research. Our analyses demonstrated that anastrozole was not as effective as letrozole in inhibiting bone age, but the high inhibitory potency of letrozole resulted in a gradual decreasing trend of HV. Anastrozole could maintain a high level of HV during treatment; thus, anastrozole was able to increase AHt more efficiently than letrozole. This supports the notion that maintaining a positive balance between adolescent growth and epiphyseal maturation can determine whether the extent of AHt can be increased, which also explains why the combination of anastrozole and rhGH exhibited the best growth effects, rather than letrozole.

In our study, we also compared the safety of letrozole and anastrozole treatment over a long period. Anastrozole and letrozole are nonsteroidal inhibitors that block androgen metabolism by reversibly binding to catalytic sites in aromatase (23, 24); the inhibitory effects of anastrozole and letrozole are approximately 97% and 99%, respectively (25). Changes in the efficacy of enzyme inhibition can lead to significant differences in the frequency and severity of adverse events (26). In this study, we found that during treatment of combined AI and rhGH, 32.8% of children exhibited hyperandrogenism manifestations such as increased body hair, a rough and greasy face, severe acne, and irritability. The anastrozole group (25.0%) had a significantly lower incidence of hyperandrogenism manifestations than the letrozole group (40.6%). The excessive suppression of estrogen by AIs leads to an increase in gonadotropin levels, which further promotes the levels of testosterone. Furthermore, letrozole can induce higher levels of gonadotropins than anastrozole (27). This increase in testosterone levels can further lead to hyperuricemia (17.2%), which can create a serious psychological burden for children. To reduce adverse reactions, our study added oral spironolactone (50-100 mg/day) to improve the symptoms of hyperandrogenism. Spironolactone is a peripheral antiandrogen drug that acts as an effective competitive inhibitor of dihydrotestosterone at the skin androgen receptor to reduce the secretion of sebum and thereby improve acne (28). After terminating AI treatment about 3.82 ± 2.94 months, the symptoms of hyperandrogen disappeared and blood testosterone returned to normal. In the present study, 2 children reported sleep problems, such as drowsiness or difficulty falling asleep, and 9 children reported mental symptoms, such as memory loss and anxiety. According to previous literature, cognitive function and risk-taking behavior may be affected by AI treatment (29). Previous studies have also reported the detection of numerous androgen and estrogen receptors in the central nervous system (30); letrozole has been reported to readily penetrate the blood-brain barrier of primates (31). This phenomenon may be the cause of adverse neurological reactions in patients receiving AI treatment. In the present study, 11 children experienced symptoms of joint pain during treatment: 8 cases in the combined AIs treatment group and 3 cases in the combined GnRHa treatment group. rhGH treatment may be associated with a reduction in bone mineral density after accelerated growth (32, 33); this may be the mechanism underlying the development of joint pain. The pathogenesis of joint pain during AI treatment is also considered to be related to a reduction in estrogen levels. Estrogen can activate endogenous neurotransmitter β-endorphin and participate in pain regulation (34). The application of AI, either directly or indirectly, can reduce the level of estrogen in the nervous system and increase the sensitivity of the body to pain. In our long-term follow-up evaluation, the abnormal monitoring indicators of the AIs group gradually returned to normal following the termination of treatment. However, it remains unclear whether AIs have long-term effects on children’s cardiovascular and reproductive systems, and further large-sample follow-up studies are now needed.

This study reviewed 96 adolescent boys with ISS. The relatively large sample size and the propensity score matching design strengthened the study's conclusions. The primary limitations of the study include the nonrandomized design and lack of a control group receiving only rhGH treatment. Furthermore, subject dropout could lead to potential selective loss, especially in the GnRHa group, which could influence the accuracy of data. Future studies should conduct multicenter randomized controlled trials to make this study more powerful.

## Conclusion

The combination of rhGH with third-generation AIs was more effective in terms of improving AHt in adolescent males with ISS and a bone age ≥ 13 years. Our follow-up analysis revealed that most adverse reactions were mild and either disappeared or were alleviated after stopping letrozole or adding spironolactone. Anastrozole was significantly better than letrozole in terms of improving AHt and was associated with fewer adverse reactions. For children with ISS in the late stage of puberty, the HV of the GnRHa group remained at a relatively low level, and GnRHa must be combined with rhGH treatment for at least 2 years to induce a significant increase in AHt. Clinicians should consider the child's individual characteristics and weigh the benefits of various treatments to select the optimal strategy.

## Data Availability

The datasets generated and/or analyzed during the current study are not publicly available because Tongji Hospital of Huazhong University of Science and Technology owns the data. However, the datasets are available from the corresponding author on reasonable request.
