# Correction to: “The Combination of Aromatase Inhibitors and GH Treatment for Idiopathic Short Stature in Male Adolescents”

**DOI:** 10.1210/clinem/dgaf358

**Published:** 2025-06-19

In the above-named article by Cui Y, Zhang Q, Hou L, and Luo X (*J Clin Endo Metab*. 2025; doi: 10.1210/clinem/dgaf271), there was an error in the Discussion section.

In the originally published article, in the Discussion section, in the fourth paragraph, the sentence “In the present study, 11 children reported sleep problems, such as drowsiness or difficulty falling asleep, and mental symptoms, such as memory loss and anxiety” was incorrect. In the corrected article, “11 children” has been updated to “2 children.” In addition, “9 children reported” has been added to the middle of the sentence. The new sentence reads, “In the present study, 2 children reported sleep problems, such as drowsiness or difficulty falling asleep, and 9 children reported mental symptoms, such as memory loss and anxiety.”

In the originally published article, in the Discussion section, in the fourth paragraph, the sentence “In the present study, 8 children experienced symptoms of joint pain during treatment: 7 cases in the combined AIs treatment group and 1 case in the combined GnRHa treatment group” was incorrect. In the corrected article, “8 children” has been updated to “11 children,” “7 cases” has been updated to “8 cases,” “1 case” has been updated to “3 cases,” and the sentence now reads, “In the present study, 11 children experienced symptoms of joint pain during treatment: 8 cases in the combined AIs treatment group and 3 cases in the combined GnRHa treatment group.”

The article has been corrected online.

doi: 10.1210/clinem/dgaf271

